# Medical and Philosophical Intelligence

**Published:** 1817-12

**Authors:** 


### MEDICAL AND PHILOSOPHICAL INTELLIGENCE.

### *Her Royal Highness the Princess Charlotte.*

THERE is a certain court etiquette which prevents an authenticated account after the demise of an illustrious female. This is not confined to the royal family:—when the late Duchess of Devonshire died, the examination of the body was delivered, sealed, to her widowed consort. Like most other secrets, however, the important events gradually transpire; and, though for the reason above-mentioned, we can plead no direct authority, yet the various sources from which the whole of the following history has been confirmed, are sufficient to satisfy us that they are generally true. Nor does it lessen the validity of our report, if, after all the circumstances we have collected, the cause of the fatal issue should not be perfectly ascertained. Every medical man is aware that the same difficulty occurs daily, more commonly in the most elevated ranks. It was an observation of the learned and ingenious Dr. Denman, that the inferior animals suffer less by parturition; and that females, the nearer they approach to a state of nature, for the most part suffer the least. A lively illustration he offers of this, in the difference which the Egyptian midwives remarked between those females about the court of Pharoah, and the Israelitish women in a state state of bondage.—Let us now consider the situation of her Royal Highness. Just relieved from all the trammels of state, and from the apprehension of an union repugnant to her wishes, and even associated with the further apprehensions of expatriation, she found herself united to the husband of her choice,—a felicity rarely experienced by females, and least of all in that exalted rank. At first a temporary gloom prevailed, lest her own hopes, the hopes of her husband, of her family, and of the empire, should be disappointed. At length she became "as those who love their lords would wish to be;" and, as this became more and more confirmed, all her prospects of earthly happiness seemed completed. Retired from the busy world, she had leisure to indulge this happiness in all its fulness. Her whole society, the females of her choice; and, when she thought proper, the intercourse of one to whose presence on an approaching period it was desirable that she should be familiarised. To complete this felicity, the residence was on a spot rendered classical by two of our celebrated poets in their best performances. The beauties of Eshur and of the Mole, (report informs us,) had been selected, to embellish a present to her husband; and, in the morning exercises, near the banks of the Thames, how often must she have reminded her companion of those lines which prove that the language she was teaching him is susceptible of all the music, if not of all the softness, of the Italian.

"Oh! could I make thy sweetly-flowing stream
"My bright example, as it is my theme,
"Though deep yet clear, gentle yet not dull,
"Strong without rage, without o'erflowing full."

Such appears to have been the uninterrupted tenor of a life too felicitous perhaps to be permanent in this transitory state of existence. It seems to bear a resemblance to that preternatural state of health from which the great father of physic teaches us to apprehend so much. But this is not all:—The whole period of gestation and parturition, it is well known, is a state of preternatural power and action. It is not difficult to guess what it must have proved with these additional excitements. We have reason to believe, though we know nothing from authority, that pains were taken to repress as much as possible a morbid excess of animal spirits, the effects of which were apprehended: but it is well known, that this is not only out of the power of the physician, but often of the patient herself.

Her Royal Highness may be said to have been fifty hours in labour, but with no dangerous symptoms, not being confined to bed during the greater part of that time. At length, the slow progress induced Sir Richard Croft to wish the sanction of another physician-accoucheur, probably lest it should become doubtful whether instruments should be used. Dr. Sims arrived about two o'clock on Wednesday morning, (Nov. 5,) and from that time the intercourse between him and Sir Richard was perpetual; but nothing occurred in the opinion of either to justify any thing beyond the ordinary means. The length of time, and other events, induced the apprehension of a stillstill-born child; and, under this impression, the necessary apparatus for re-animation was in readiness.

At nine on Wednesday evening, her Royal Highness was delivered of a still-born child, which, as far as we can learn, Drs. Sims and Baillie were endeavouring to re-animate, whilst Sir Richard remained with the royal mother. During the whole period, and for some time after, no unfavourable symptom occurred, excepting that her Royal Highness was less exhausted than might have been expected by so tedious a labour and the subsequent events. Sir Richard, suspecting the hour-glass contraction from the tediousness of the subsequent process, thought it right to give some assistance, having of course first consulted and obtained the concurrence of his coadjutors. All this was accomplished without difficulty, and with no apparent danger, excepting what arose from the almost unnatural composure, not to say cheerfulness, of her Royal Highness.

In this manner things remained for nearly three hours after the birth. At this time her Royal Highness was sick, and threw up part of a cardiac medicine she had taken; and, with the advice of Drs. Baillie and Sims, we have heard that an opiate was administered. Her Royal Highness remained quite composed for some time after this, and got some sleep; but about a quarter before twelve great restlessness came on; and Sir Richard Croft found it necessary to call in the other physicians. From that time the fatal issue advanced rapidly; a slight difficulty in swallowing, which soon subsided, added to the sickness, was all that had previously occurred. But from this time pain in the chest, great difficulty in respiration, and extreme restlessness, increased, with a rapid, irregular, and weak pulse, till the vital spark was extinguished. It is scarcely necessary even to hint, that every means of support was administered. At two o'clock, on Thursday morning, her Royal Highness ceased to breathe!

The appearances after death are pretty well known. On examining the contents of the cranium, the dura mater was found natural, the vessels of the pia mater were less loaded with blood than often occurs, and the plexus choroides somewhat pale; in the ventricles was a small quantity of water; the substance of the brain natural. The pericardium contained two ounces of red fluid. The stomach a good deal of fluid, probably most of what had been taken after the sickness. The abdominal viscera were quite natural. The uterus was so little contracted as still to reach as high as the umbilicus; the hour-glass form was still apparent, it contained a considerable quantity of coagula within its cavity,—from what we can learn, about a pound.

To what then are we to impute a death which has filled the whole nation with distress? A labour much longer protracted has often ended happily for mother and child; the slow contraction of the uterus, however unfavourable in itself, was unattended with any consequences which should excite alarm. The fluid in the pericardium might readily explain the severe pain in the chest, the irregularity of the pulse, and might even prevent the heart from recovering its vigorous action. Was this extravasated during the pains, and were the consequent sensations suspended for a time by the composure of the royal patient, during so long, so tedious, and without doubt often so painful, a labour? Where all is conjecture, we may be allowed to offer ours.

---

*To the Editors of the London Medical and Physical Journal.*

GENTLEMEN,

SOME time ago you were pleased to notice, in terms of the most flattering approbation, my humble efforts to improve operative surgery by the invention of my "Annular Saw." This instrument has obtained so generally the sanction of the most eminent surgeons in the metropolis, and in various parts of the country, as to become part of the surgical apparatus in the depots of the army and navy, and in most of the provincial hospitals.

From a fondness for the mechanic sciences, I have directed much of my attention to their application in surgery; and have considered, with the closest attention, the nature and treatment of those diseases, which, being marked by a greater or less deviation from the ordinary symmetry of parts, are included under the general term Distortion, whether arising from original malconformation, muscular contraction, debility, or the effects of accidental violence. Much reflection and experience convince me, that the mode in which adventitious aid is usually applied in cases of this description, has arisen from erroneous pathology, and is, consequently, for the most part, very inadequate to the cure.

I am now substituting, in the place of metallic inelastic machines, the inefficacy of which the generality of practitioners of the present day admit, an apparatus which, whilst it affords the necessary support, is constructed in such a manner as incessantly to counteract the morbid inclination, or unnatural deflection, of the parts, without occasioning painful weight, pressure, or any unsightly appearance. I have the honour to be, &c.

THOMAS MACHELL,
Member of the Royal College of Surgeons.

*Great Ryder-street, St. James's;*
*Sept.* 1817.

*** We are glad to find that this most ingenious artist has in the press a practical Treatise illustrative of his improved mode of treating Distortions in general; and, most of all, that he has been prevailed on to direct his chief attention to a department of surgery which has hitherto been consigned to individuals wholly unacquainted with the principles and practice of medical science, the more immediate, and originally the sole object of his professional exertions.—EDIT.

---

In our last, we mentioned a *fracas* between a royal physician and a surgeon of some experience, almost over the dead body of Mr. Curran, the late Irish Demosthenes. The surgeon, having now left the country, may be mentioned by name. Mr. ROBERTON, whose correspondence has lately appeared in our Journal, contrived to

 elude elude the diligence of the police-officers, till he embarked to assume the head of the medical staff of the insurgent army in South America. This appointment is well suited to one not deficient in abilities or practical knowledge. It is also no inconsiderable acquisition in a country to which few at his age would be willing to migrate.

---

A late event has proved, that the venom of a viper, when applied in a sufficient quantity, or to a sufficient surface of absorption, is equal to produce death in a full-grown human subject. A confidential servant of a nobleman was amusing some of the younger part of the family, by exhibiting the apparatus for the venom in a dead viper. Unfortunately, the man's fingers had been excoriated, and the application of the poison proved fatal on the following day. This is the second authenticated case which we can offer of death from such a cause. In all but one other instance which have come to our knowledge, (three in number,) the limb in which the puncture was inflicted, has become extremely tumid, and the skin over the whole body suffused with yellow: some fever has attended, but in three or four days the patient recovered.

The other fatal case was in a practitioner in Essex. Retiring from the high road *to uncover his feet,* as is delicately expressed in holy writ, he trod on a viper, which instantly bit him in the scrotum. Whether from the delicacy of the part, and, possibly, from the wound penetrating to the testicle, the symptoms increased in severity till death. It need scarcely be added, that every means of assistance was soon at hand.

---

*St. George's Hospital.*—Sir EVERARD HOME removed lately, from the head of a woman, a tumour weighing fifteen pounds and some ounces. It consisted of fat attached to a basis of bone, which was taken away by sawing it from the cranium. There was not so much hæmorrhage as might be expected, though the operation was nearly an hour performing. The patient is doing very well.

---

*Death by Lightning.*—During a visit to a friend in Herefordshire, I was desired to examine the body of a man whose life had been suddenly destroyed by lightning, at Colwall, near Ledbury. The wife of the deceased obstinately refusing permission to open the body, my examination was confined to the effects of the electric fluid on the external surface. On viewing the head, I found the hair and the beard of the left side singed, and the skin of the ear, cheek, and upper part of the neck, perfectly black, but entire. Between the shoulders, there was another black spot of the size of a crown, exactly over the spine, and on the outside of the thigh of the right side just above the knee, there was another black spot, which I could scarcely cover with my hand. The parts of the shirt and flannel lining of the breeches and jacket which covered the injured skin were charred, but neither the exterior parts of the small-clothes (which were made of corduroy) nor of the jacket, were burnt, the electric fluid having only occasioned a laceration resembling an incision made by a sharp instrument. It appears, that the electric matter entered the left side of the head, passed through the chest

3 X 2 and and abdomen in an oblique direction, and escaped just above the knee on the opposite side of the body. Whether the fluid entered or escaped at the spot on the back, I am at a loss to say; but from the external part of the jacket not being burnt, I suspect a quantity escaped there. The spots were perfectly black, and exhibited the same appearance as is produced by the caustic alkali after remaining several hours on the skin; and, from its flabby state, the mischief was no doubt deep. Whether the fluid produced the same effect on the internal parts through which the fluid passed as it did on the skin, is a question which I shall be obliged to you, or some of your readers who have ascertained the fact in a similar case, to answer. The man, at the time the accident happened, was under an oak-tree, and, when the lightning struck him, he sprung forward and fell on his face; soon after which, there was a second flash, and this might have produced the spot between the shoulders; but if so, where did it escape?

About twenty years ago, I had an opportunity to examine a man who was struck dead by lightning in a field near Hereford, with an umbrella over his head. On that man the electric fluid produced no evident effect, either externally or internally. The brain had a sulphurous smell.

*Queries.*—Did the passing of the fluid through the umbrella prevent its burning the body? As no apparent injury was done to the body, how are we to account for its effects in destroying life? Did it terminate life by destroying the electrical powers of the brain? I hope some of your readers will, through the medium of your valuable work, favour me with some remarks on these cases, and replies to my queries. R. R.—*Phil. Mag.* ———

*Optics.*—A very interesting case has just occurred, of a person born blind being restored to sight by the means of a surgical operation:—A native of Burdwan, of the age of eighteen, was lately sent by his family to Dr. Luxmore, of whose success in the removal of the cataract they had heard by public report. The operation was performed on the 26th, and in six days he began to see and distinguish objects. After the celebrated case of Dr. Cheselden's patient, whose sensations have been so minutely and philosophically laid before the public, it can hardly be expected that any discovery regarding the origin of our ideas of figure, distance, or quantity, could be extracted from the observation of an ignorant country boy, who, unaccustomed to think abstractedly, is little able to describe the gradual improvement of his intellect, under this sudden and astonishing introduction to the visible world. He confirmed, however, with readiness the conclusion, so obvious from the feelings of Dr. Cheselden's patient, that our common judgment of figure, quantity, and distance, is not an inherent faculty in the mind, but a practical result, from the ever-repeated experiment of comparing the perspective with the actual figure, bulk, or distance. For a cricket-ball was put in one hand, and a cube of soap in the other, and he was desired to describe their shape; he was unable to do it by his newly-acquired and inexperienced vision, and was obliged to have constant recourse to the more practised sense of feeling. When any object ject is presented to him, although he can, without hesitation, declare its colour, he is wholly unable to decide on its quality, until he is allowed to handle it.—*Bengal Paper.*

---

*Hybernation of Swallows.—Extract of a Letter from Joseph Wood, Esq. to a Gentleman in Washington, dated Marietta, June* 30.

I came to this country in the autumn of 1785, and resided at Belleville, about three miles below this place, on the Virginia side, till 1791. During my residence there, I observed one evening, a little after sunset, a vast number of swallows collected together high in the air, and hovering over a particular spot; this was in autumn, when the weather began to grow cool. Having been informed by some of my school-mates, when a boy, that they had seen swallows dive into a mill-pond, and disappear; I was determined to watch these, and in about ten or fifteen minutes, as darkness approached, they lowered their flight, and concentrated in a smaller circle, and at length, to my surprise, poured into a very large hollow sycamore-tree, about seventy feet above the ground. I observed that they came out for several successive days, and returned in the evening in the same manner. In the following year, some of the settlers cut down the tree; the hollow was about six feet in diameter, and was filled six inches deep with bones and feathers, and other remains of dead birds;—such, probably, as were too old and feeble to fly out in the spring. They must have occupied the tree for many years. I have since seen two other trees that have fallen, with similar appearances.—*Phil. Mag.*

---

Our readers, who are not ignorant of the strong parties which exist in Liverpool concerning Miss M'Evoy, may consider the following as a hoax of the incredulous sect. We know no information beyond what we meet with in the Times Newspaper.

"An extraordinary discovery in natural history was made at Liverpool about a fortnight ago. As one of the stone-masons, in the employ of the Dock Trustees, was dressing, on the sea-wall of the Regent's Dock, a huge stone, brought from the Western Point quarry, and after he had broken a considerable thickness from its outside, he discovered, in a hole of small diameter which was partially filled with clay and a loamy sand, three bees, in a state of animation, to the inexpressible astonishment of himself and fellow-workmen, many of whom were witnesses of this strange phenomenon. The foreman of the works put them into his handkerchief, where they remained for several hours afterwards; but, while exhibiting his newly-resuscitated strangers, two of them flew away, and he voluntarily gave the third its liberty. These bees are described to us as having been of the drone species. We have questioned the person as to the truth of so singular a statement, and he affirms, that they were found in the interior of the solid stone, as we have described above, without any perceptible communication from without. Toads, and other similar animals, have been found, in a living state, in situations equally extraordinary; but we never heard before of any of the winged tribe being incased in the heart of a solid stone. This discovery is singular, and will furnish matter of curious speculation to the naturalist and the philosopher."

### REPORT OF DISEASES.

The following circular was, we believe, sent to most of the hospital and dispensary physicians in and about the metropolis:—

Gentlemen,—The Medical Committee of the Institution for the Cure and Prevention of Contagious Fevers in the Metropolis, being desirous of laying before the public the best information which they can procure relative to the present state of febrile diseases in London, take the liberty of requesting you to favour them with your opinion as to the following points, viz.—Whether there has been, within the last few months, a greater quantity of fever admitted than for some time previous; and if so, of what character it has been, and of what degree of severity?

The Committee having adjourned from this day to Tuesday next, the 11th inst. they will feel themselves particularly obliged by the favour of an answer, forwarded to me by Monday next, the 10th inst.

I am, Gentlemen, your obedient humble servant,

13, *John-street*, *Bedford-row*; (For Charles Murray, Secretary,)
*Nov.* 4, 1817. James A. Murray.

It may seem strange that such a circular should appear at a time when the town is actually healthier than usual. That the late distresses have occasioned the usual fever attendant on want of food, and on other privations, is beyond a doubt; but, it is equally certain, that this fever has been less frequent and milder among the poor, than that which occurred at the close of the last, or beginning of the present, century. The reason is obvious,—the distresses have been less, and the readiness at meeting them by subscriptions greater. The want of employment, too, has been more temporary, and the principal source of complaint now is the low rate of wages, particularly for common and agricultural labour. As hands are more wanted, this evil, it is to be hoped, will cure itself. Those who see as much of the working part of the community as falls to the lot of medical men, must be aware that the imputation of idleness is far more general than the fact authorizes. The truth is, that most of these people are disposed to work too hard. It must be acknowledged, that this excess of labour often induces, whilst it serves as an apology for, an excess of drinking, both of which are inconsistent with health or longevity. On all these occasions, the fault is as often with the employer as with the labourer. Men, properly educated, must, we should conceive, be aware, that, as a horse, however well fed, is soon worn out by hard work, the condition of a man must be similar. It becomes, therefore, the duty of masters to refuse an improper share of labour, and to employ more hands, rather than indulge this disposition to support unnatural exertions by artificial means. When men cannot continue their work without having recourse to an immoderate quantity of porter, no arguments are required to prove, that they are destroying themselves by labour, at the same time that a considerable part of their earnings is expended in increasing the very means of destruction.

All this while, our readers may be expecting us to say something about *typhus* If any thing could increase the absurdity of the term, it is, that at last it has reached Prince Leopold.—He, we are told, has had symptoms of typhus. If, as the nosologists teach us, typhus is *febris lenta nervosa*, certainly he has had cause enough for such an effect; and, if the term is confined to such cases, or to any cases whatever, we might learn what those who use it mean. That the fever from poverty has appeared in a mild form among the labouring class, cannot be questioned, and that it has, by some accidental communication, reached the mansions of the rich, is equally certain. But, in the first instance, it has extended to the whole house, and often to the whole neighbourhood; in the last, it has ceased with the person affected. It is true, in the latter instances, the disease has been more severe, and sometimes fatal. This has occurred oftener in Ireland, and the provincial towns of England, than in London, because the distress having been greater, the quantity of fomes inhaled in the same quantity of atmosphere has been much more considerable, and, when received by a person in high health, and well fed, the inflammatory symptoms have been great in proportion.

The mildness of the season, in other respects, has lessened the severity of the winter asthmas and coughs. The remains of diarrhœa and dysentery continue, but in a chronic form.

Most of the other complaints are inflammatory. In all, excepting pleurisy and other inflammations of the membranes which line cavities, topical bleeding has been universally serviceable. In many, brisk cathartics have been sufficient. Blistering is never serviceable till the disease has become completely chronic.

Small-pox, and the other contagions, are not particularly prevalent, and for the most part mild.

### A METEOROLOGICAL JOURNAL,

By Messrs. William Harris and Co. 50, Holborn, London.

*From the 20th October to the 19th November, inclusive.*

| Day of Month. | Moon. | Rain gauge | Therm. | | | Barom. | | De Luc's Hygrom. Dry | De Luc's Hygrom. Damp | | Wind. | | Atmospheric Variation. | | |
|---|---|---|---|---|---|---|---|---|---|---|---|---|---|---|---|
| Oct. | | | | | | | | | | | | | | | |
| 20 | | | 45 | 47 | 44 | $29^{9}$ | $29^{9}$ | | 1 | 1 | N | NNE | Rain | | Clo. |
| 21 | | | 47 | 49 | 40 | $29^{9}$ | $29^{8}$ | | 2 | 2 | NE | E | Fog. | Rain | Clo. |
| 22 | | ,05 | 43 | 50 | 41 | $29^{8}$ | $29^{8}$ | | 2 | 1 | N | N | Fog. | Clo. | |
| 23 | | | 45 | 48 | 41 | $29^{8}$ | $29^{8}$ | | 1 | 1 | NNE | NE | Fine | | Clo. |
| 24 | | ,09 | 44 | 52 | 44 | $29^{8}$ | $29^{8}$ | | 2 | 1 | N | SE | Fog. | Clo. | Rain |
| 25 | ○ | ,08 | 44 | 50 | 41 | $29^{8}$ | $29^{7}$ | | 2 | 2 | SE | S | Fine | | |
| 26 | | | 45 | 49 | 38 | $29^{8}$ | $29^{8}$ | | 2 | 2 | S | S | Fine | | |
| 27 | | ,06 | 44 | 47 | 40 | $29^{9}$ | $29^{9}$ | | 1 | 2 | S | W | Fog. | Clo. | Rain |
| 28 | | ,04 | 46 | 52 | 40 | $29^{8}$ | $29^{6}$ | | 1 | 2 | SSE | SE | Fog. | Fine | Clo. |
| 29 | | ,09 | 45 | 49 | 40 | $29^{6}$ | $29^{6}$ | | 2 | 1 | S | SW | Rain | | |
| 30 | | ,13 | 40 | 47 | 41 | $29^{4}$ | $29^{4}$ | | 2 | 1 | SSW | WSW | Rain | | |
| 31 | | ,19 | 40 | 48 | 38 | $29^{3}$ | 30 | | 2 | 2 | W var. | WSW | Clo. | Rain | Fine |
| Nov. | | | | | | | | | | | | | | | |
| 1 | | | 42 | 48 | 34 | $30^{3}$ | $30^{5}$ | | 2 | 2 | SW | S | Fog. | Fine | |
| 2 | ☽ | | 45 | 47 | 49 | $30^{3}$ | $30^{2}$ | | 3 | 3 | S | SSW | Clo. | | Rain |
| 3 | | ,09 | 54 | 57 | 50 | $30^{2}$ | $30^{1}$ | | 5 | 6 | S | SE | Clo. | | Fog. |
| 4 | | | 50 | 53 | 48 | $30^{1}$ | 30 | | 6 | 5 | SE | SE | Clo. | | |
| 5 | | | 50 | 55 | 50 | $29^{9}$ | $29^{9}$ | | 5 | 5 | SSE | SE | Fog. | Clo. | |
| 6 | | | 52 | 54 | 52 | $29^{9}$ | $29^{8}$ | | 6 | 6 | SE | SE | Clo. | | Rain |
| 7 | | .11 | 55 | 57 | 49 | $29^{7}$ | $29^{5}$ | | 5 | 5 | SSE | S | Fine | | Rain |
| 8 | | .07 | 50 | 51 | 47 | $29^{5}$ | $29^{5}$ | | 3 | 3 | S | S | Fine | | Rain |
| 9 | ● | ,13 | 49 | 52 | 50 | $29^{7}$ | $29^{9}$ | | 2 | 2 | SW | S | Fine | | |
| 10 | | | 52 | 53 | 49 | $29^{9}$ | $29^{9}$ | | 2 | 2 | S | SSE | Clo. | Rain | Clo. |
| 11 | | ,07 | 51 | 54 | 49 | $29^{9}$ | $29^{8}$ | | 2 | 1 | SE | S | Fine | Rain | Fine |
| 12 | | ,10 | 50 | 52 | 45 | $29^{7}$ | $29^{5}$ | | 1 | 5 | S | S | Clo. | Rain | |
| 13 | | ,19 | 45 | 50 | 47 | $29^{8}$ | $29^{7}$ | | 4 | 2 | SW | ESE | Fine | | |
| 14 | | | 52 | 55 | 49 | $29^{7}$ | $29^{5}$ | | 2 | 3 | SE | SE | Clo. | Rain | Fine |
| 15 | ☾ | ,03 | 50 | 58 | 50 | $29^{5}$ | $29^{6}$ | | 5 | 2 | S | S | Rain | | |
| 16 | | ,09 | 50 | 53 | 47 | $29^{8}$ | $30^{1}$ | | 2 | 5 | S | SW | Clo. | Rain | Clo. |
| 17 | | | 49 | 57 | 45 | $30^{2}$ | $30^{2}$ | | 7 | 8 | SW | SW | Clo. | Rain | |
| 18 | | | 49 | 53 | 45 | $30^{2}$ | $30^{2}$ | | 5 | 5 | SSW | WSW | Fine | | |
| 19 | | ,05 | 47 | 54 | 39 | $30^{3}$ | $30^{4}$ | | 4 | 4 | W | W | Fine | | |

The quantity of rain fallen in the month of October is 1 inch and 65-100ths.

